# Intention to vaccinate universally against varicella, rotavirus gastroenteritis, meningococcal B disease and seasonal influenza among parents in the Netherlands: an internet survey

**DOI:** 10.1186/s13104-017-3004-z

**Published:** 2017-12-04

**Authors:** Alies van Lier, José A. Ferreira, Liesbeth Mollema, Elisabeth A. M. Sanders, Hester E. de Melker

**Affiliations:** 1 Centre for Infectious Disease Control, National Institute for Public Health and the Environment (RIVM), PO Box 1, 3720 BA Bilthoven, The Netherlands; 2 Expertise Centre for Methodology and Information Services, National Institute for Public Health and the Environment (RIVM), PO Box 1, 3720 BA Bilthoven, The Netherlands; 3 Department of Pediatric Immunology and Infectious Diseases, Wilhelmina’s Children Hospital, University Medical Center Utrecht (UMCU), PO Box 85090, 3508 AB Utrecht, The Netherlands

**Keywords:** Varicella, Rotavirus, Meningococcal B disease, Seasonal influenza, Vaccination, Acceptance, Epidemiology

## Abstract

**Objective:**

For the decision-making process regarding introduction of new vaccines into the National Immunisation Programme (NIP), advance insight into the potential acceptance among the population is relevant. We studied the intention of parents to have their child vaccinated against four diseases not currently covered by the NIP in the Netherlands. The results on varicella have been published before; this article adds the results on vaccination against rotavirus gastroenteritis, meningococcal B disease, and seasonal influenza.

**Results:**

We invited a random sample from the national immunisation register of 1500 parents for an internet survey which was completed by 491 parents (33% response). The intention to vaccinate was highest for meningococcal B disease (83% positive intention), followed by rotavirus gastroenteritis (38%), and lowest for varicella (28%) and seasonal influenza (15%). Prediction analyses were performed to determine which out of seven questionnaire statements was most informative in predicting the intention to vaccinate. Main drivers of intention were the perceived importance of vaccination against the particular disease and the perception of whether or not the disease is severe enough to justify vaccination. The results of this study can be informative in the decision-making process whether or not to introduce new vaccines into the NIP.

**Electronic supplementary material:**

The online version of this article (10.1186/s13104-017-3004-z) contains supplementary material, which is available to authorized users.

## Introduction

Nowadays, the Dutch National Immunisation Programme (NIP) includes vaccination against twelve vaccine-preventable diseases; it is free of charge and voluntary (Additional file 1: Table S1). New vaccines are constantly under development and may become eligible for inclusion in the NIP [1]. In the Netherlands, the Dutch Health Council advises on the inclusion of new vaccines in the NIP [2]. In general, vaccines which are registered but not included in the NIP have only rarely been used in the Netherlands.

A study in 2004 observed that 11% of parents would object to having their child vaccinated with any newly introduced vaccine in the Dutch NIP [3]. Another study found that in the Netherlands, in 2003/2004, 22% of parents and 28% of Child Health Clinic professionals were of the opinion that nowadays too many vaccinations are administered to children [4]. They concluded that for most parents the severity of a disease, and not so much the frequency of occurrence of a disease, was important in their decision regarding new vaccines. However, the above mentioned studies were conducted a number of years ago, and since then new vaccines against pneumococcal disease, human papillomavirus infection and hepatitis B have been introduced which might have influenced the opinion of parents regarding introduction of new vaccines.

For the decision-making process regarding the introduction of new vaccines into the NIP, advance insight into the potential acceptance among the population is relevant. Moreover, objections against new vaccines may harm the high vaccination coverage of vaccines already included in the NIP. And for vaccination against some diseases (e.g., varicella) high vaccination coverage to induce herd protection is important, since otherwise occurrence of varicella disease may only be pushed towards older age groups which is undesirable because of the higher risk of complications at older age. Therefore, we studied the intention of parents to have their child vaccinated against four diseases not currently covered by the Dutch NIP. The results on varicella have been published before [5]; this article adds the results on vaccination against rotavirus gastroenteritis, meningococcal B disease, and seasonal influenza.

## Main text

### Materials and methods

#### Study population and design

We selected a random sample of 1500 parents with at least one child aged 0–4 years from the national immunisation register (Præventis) [6], after approval by its registration committee. In November 2012, parents were invited for an internet survey by a letter from the National Institute for Public Health and the Environment (RIVM). After 3 weeks, parents who did not respond received a reminder. The survey contained questions on background characteristics, vaccination in general, and vaccination against varicella, rotavirus gastroenteritis, meningococcal B disease, and seasonal influenza (see Additional file 2). Respondents were asked to rate the severity of different diseases on a scale from 1 (not severe at all) to 10 (very severe) because previous research showed that disease severity was important for decision-making on new vaccines [4]. We included seven statements to measure intention, attitude (general, and two attitude-related constructs: risk perception and outcome expectation) and subjective norm (see Additional file 2: questions 18–23). These statements were selected based on the results of another questionnaire developed for a study on introduction of vaccination against hepatitis B [7] using the Theory of Planned Behaviour [8]. In this study, perceived behavioural control failed to explain any unique variance in intention [7] and therefore we did not include it. The level of agreement on statements was measured using a 5-point Likert scale.

#### Data analysis

For the intention to vaccinate, we calculated the mean score as well as the percentage of parents with a positive intention (i.e., parents who would ‘*definitely*’ or ‘*probably*’ vaccinate their child). To find out whether there are differences in intention (mean score) between vaccination within the NIP free of charge and self-payed vaccination outside the NIP, paired-sample *t*-tests were conducted.

In order to determine which out of seven statements in the questionnaire was most informative in predicting the intention of parents to vaccinate their child, prediction analyses were carried out by disease using randomForest software [9]. A randomForest is an algorithm that predicts the outcome (intention to vaccinate) of an individual on the basis of the individual’s predictor variables (the answers to the questionnaire statements). RandomForest assesses the importance of a predictor variable by determining how much the prediction error increases (i.e., accuracy decreases) as a result of random permutation of the data on that variable while the data on the other variables are left unchanged. If a variable does not contribute to the prediction of the outcome then the error estimates based on the original dataset are about the same as those based on the dataset where the variable in question has been randomly permuted. On the contrary, the prediction errors will increase by random permutation of its values whenever a variable is crucial in predicting the outcome. For these prediction analyses the intention to vaccinate was divided into three categories: (a) positive intention (‘*yes, definitely*’ or ‘*probably yes*’), (b) neutral (‘*neutral*’), and (c) negative (‘*no, never*’ or ‘*probably not*’).

Data analyses were performed in SPSS (version 22.0) and R.

### Results

The survey was completed by 491 parents (33% response). Background characteristics of the respondents and some results regarding varicella have been described previously [5]. Most parents had a positive opinion on vaccination in general: they felt that vaccinating their child is a matter of course (78%), and were of the opinion that childhood vaccinations are good to protect their own child’s health (92%) as well as for the protection of others (66%) (Additional file 1: Figure S1). Some of the parents (11%) had the feeling that too many vaccinations were administered to children nowadays.

#### Ranking perceived severity diseases

Most diseases against which vaccinations are currently included in the NIP were perceived as being (very) severe as the mean rating was above 7 except in the case of measles, mumps and rubella (Additional file 1: Figure S2). Parents also perceived meningococcal B disease (mean rating 8.6) as a very severe disease, while seasonal influenza (3.7) and varicella (4.1) were seen as being relatively mild; rotavirus gastroenteritis (7.2) scored in-between.

#### Intention, attitude and subjective norm

**Fig. 1:**
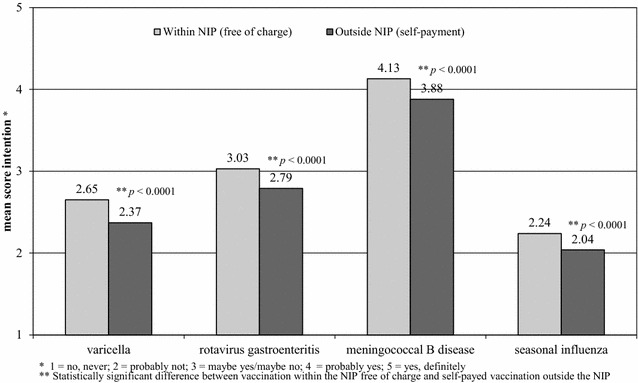
Intention (mean score) of parents to vaccinate their child against varicella, rotavirus gastroenteritis, meningococcal B disease, and seasonal influenza if the vaccine is included in the National Immunisation Programme (NIP) free of charge versus self-payment outside the NIP

The intention to vaccinate was highest for meningococcal B disease (83% positive intention), followed by rotavirus gastroenteritis (38%), and lowest for varicella (28%) and seasonal influenza (15%). For each disease, the intention to vaccinate (mean score) was somewhat lower if parents were to be charged for the vaccination (*p* < 0.0001) (Fig. 1).

**Fig. 2:**
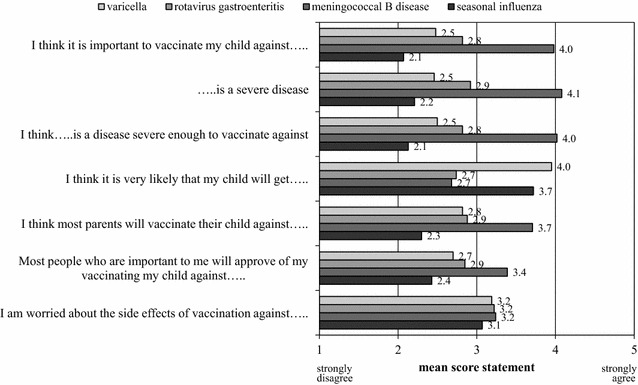
Opinion of parents on statements (mean score) regarding vaccination against varicella, rotavirus gastroenteritis, meningococcal B disease, and seasonal influenza

Meningococcal B disease was the most important disease to vaccinate against according to the participating parents, and seasonal influenza and varicella the least important, while rotavirus gastroenteritis scored in-between (Fig. 2). Meningococcal B disease was perceived as a severe disease, and severe enough to vaccinate against, in contrast to seasonal influenza and varicella. Although the respondents thought that it is not very likely that their child will contract meningococcal B disease, they thought that most parents will vaccinate their child against it and that most people who are important to them will approve of their vaccinating their child against it. Regarding rotavirus gastroenteritis, varicella and especially seasonal influenza, the agreement with these statements was much lower in spite of the perceived higher likelihood of their child contracting varicella or seasonal influenza. With regard to concerns about side effects of vaccination, the results per disease were more or less the same. Half of the parents (53%) liked the rotavirus vaccine being orally administered.

#### Prediction analyses

**Fig. 3:**
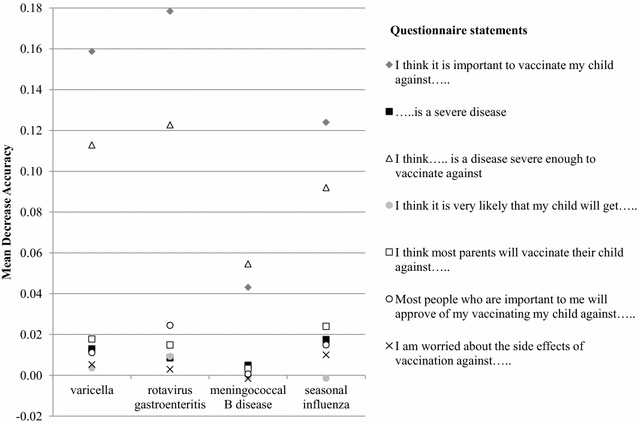
Importance of questionnaire statements in predicting the intention to vaccinate (divided into positive, neutral or negative intention) against varicella, rotavirus gastroenteritis, meningococcal B disease, and seasonal influenza. The Mean Decrease in Accuracy of a given predictor variable (questionnaire statement) is the decrease in the proportion of correct predictions regarding the outcome (intention to vaccinate) that results from randomly permuting the values of that variable in the dataset

The randomForest algorithm predicted intention to vaccinate correctly in 72.5% (varicella), 74.7% (rotavirus gastroenteritis), 85.9% (meningococcal B disease), and 76.6% (seasonal influenza) of the time. The following statements had the greatest predictive value for the intention to vaccinate: ‘*I think it is important to vaccinate my child against…..*’ and ‘*I think….. is a disease severe enough to vaccinate against*’ (Fig. 3).

### Discussion

Before adding new vaccines to a NIP, it is important to have advance knowledge about the intended acceptance by the population. The intention of parents to have their child vaccinated was relatively high for meningococcal B disease (83% positive intention), intermediate for rotavirus gastroenteritis (38%), and low for varicella (28%) and seasonal influenza (15%). For meningococcal B disease, most participating parents (82%) thought the disease to be severe enough to prefer vaccination, while for seasonal influenza and varicella most parents stated that the disease is not severe enough to vaccinate against (74 and 59% respectively). For rotavirus gastroenteritis the opinion was less outspoken: 44% of the parents felt that the disease is not severe enough to vaccinate against while 30% felt that it is.

Main drivers of intention in this study were the perceived importance of vaccination against the particular disease and the perception of whether or not the disease is severe enough to justify vaccination, while the perceived risk of contracting the disease was of lesser relevance, in agreement with observations by Van de Bovenkamp and Rümke [4]. Other studies also showed that willingness of parents to vaccinate against new diseases depends on the disease in question. Hak et al. observed that the proportion of parents with a positive attitude towards vaccination of children was much lower for seasonal influenza or pneumonia (both 36%) than for hepatitis B (62%), SARS (64%), tuberculosis (67%), and smallpox (79%) [3]. Van de Bovenkamp-Meijer and Rümke found that the percentage of parents with a positive intention to have their child vaccinated was much lower for seasonal influenza (22%) and varicella (39%) than for hepatitis B (71%), pneumococcal disease (93%) or meningococcal B disease (97%) [4]. Harmsen et al. found a positive intention (measured on a 7-point Likert scale) among parents to vaccinate children against meningococcal B disease (72%), rotavirus gastroenteritis (50%), varicella (43%) and seasonal influenza (22%) (unpublished data). Furthermore, Harmsen et al. showed that providers of childhood vaccinations believed that vaccines against meningococcal B disease, the respiratory syncytial virus, and rotavirus gastroenteritis were most necessary within the NIP; they perceived vaccination against varicella and seasonal influenza as less important [10].

Finally, in this study, 11% of the parents had the feeling that too many vaccinations are administered to children nowadays in contrast to 22% found in another Dutch study conducted in 2003/2004 [4].

### Conclusions

To conclude, this study showed that the intention of parents to have their child vaccinated against newly introduced vaccines in the NIP varied by disease and was mainly related to the perceived importance of vaccination against the particular disease and the perception of whether or not the disease is severe enough to justify vaccination. The results of this study can be informative in the decision-making process whether or not to introduce new vaccines into the NIP.

## Limitations

This study has some limitations. The response rate of 33% was rather low, but higher or in the same range as in other studies among Dutch parents 15% [11], 16% [12], 37% [13]. Respondents with a high education level were overrepresented, whereas respondents with at least one parent born in another country were underrepresented [5]. As highly educated parents have a more negative attitude towards adding new vaccines to the NIP [3], our results may have underestimated the intention of parents to vaccinate against varicella, rotavirus gastroenteritis, meningococcal B disease and seasonal influenza. However, in our study we found a negative association between education level and the intention to vaccinate only for rotavirus gastroenteritis. Furthermore, a considerable part of the parents in our study were indecisive regarding acceptance of new vaccines, and we know that the elicited intention can differ from actual vaccine uptake anyway [14]. For rotavirus gastroenteritis, their indecisiveness might be related to their not being familiar with the disease in question as 42% of the respondents did not answer the question on perceived severity of rotavirus gastroenteritis. Finally, we did not conduct a full study on possible determinants of intention according to the Theory of Planned Behaviour but selected seven statements to measure attitude and subjective norm based on the results of another questionnaire developed for a study on introduction of vaccination against hepatitis B [7].

## Additional files

**Additional file 1.** Background on the National Immunisation Programme in the Netherlands.

**Additional file 2.** Internet survey.

## Abbreviations

NIP: National Immunisation Programme
RIVM: National Institute for Public Health and the Environment
