# Supplementary material for: Intention to vaccinate universally against varicella, rotavirus gastroenteritis, meningococcal B disease and seasonal influenza among parents in the Netherlands: an internet survey
Source: BMC Res Notes. 2017 Dec 4;10:672. doi: 10.1186/s13104-017-3004-z (PMC5716237; doi:10.1186/s13104-017-3004-z)
Supplement: Supplementary file 1 — Additional file 1. Background on the National Immunisation Programme in the Netherlands. [file 13104_2017_3004_MOESM1_ESM.docx]

**Additional file 1 Background on the National Immunisation Programme in the Netherlands**

Since 1957, the National Immunisation Programme (NIP) is offered free of charge and voluntary to all children in the Netherlands. The Dutch NIP started with vaccination against polio, diphtheria, tetanus and pertussis (1957) and gradually expanded with vaccination against rubella (1974), measles (1976), mumps (1987), *Haemophilus influenzae* type b (1993), meningococcal C disease (2002), pneumococcal disease (2006), human papillomavirus infection (2009), and hepatitis B (2011; before 2011 risk groups were vaccinated). The current vaccination schedule is shown in Table S1. Vaccinations are administered at local level by the network of Child Health Clinics and by Public Health Services. All childhood vaccinations are registered in a national electronic immunisation register which has a link to the Dutch population register [1].

In general, participation in the NIP in the Netherlands is high (also internationally), with an uptake of about 95% for most childhood vaccines, except for HPV (53%) [2, 3]. In the Netherlands, studies have shown that the majority of parents who vaccinate their children according to the NIP perceived it as self-evident [4, 5]. More recently, Lehman et al. found that only 21% of the vaccination decisions of all parents were classified to be informed [6]. In recent years, a small decreasing trend in vaccination coverage is seen in the Netherlands; e.g., the coverage for the first MMR decreased with 2.2% (from 96.0% among birth cohort 2011 to 93.8% among birth cohort 2014) [3]. The participation among the orthodox Protestant minority in the Netherlands has always been considerably lower but seems to be increasing over time [7]. Social clustering of unvaccinated children also occurs at schools with an anthroposophical signature [8].

Recently, the Health Council proposed a new approach to vaccination care [9]. In this approach, certain vaccines that are not included in the NIP could be (partially) reimbursed by health insurance companies for selected individuals or vulnerable groups in the future. The vaccines against the diseases covered in this manuscript could be eligible for this alternative option.

Table S1 Immunisation schedule of the National Immunisation Programme, the Netherlands, 2017

| **Phase** | **Age** | **Vaccination-dose** |
| --- | --- | --- |
| Phase 1 | 0 months (< 48 hours) | HepB-0^a^ |
|  | 2 months | DTaP-IPV-Hib-HepB-1 + PCV-1 |
|  | 3 months | DTaP-IPV-Hib-HepB-2 |
|  | 4 months | DTaP-IPV-Hib-HepB-3 + PCV-2 |
|  | 11 months | DTaP-IPV-Hib-HepB-4 + PCV-3 |
|  | 14 months | MMR-1 + MenC |
| Phase 2 | 4 years | DTaP-IPV-5 |
| Phase 3 | 9 years | DT-IPV-6 + MMR-2 |
| Phase 4 | 12 years | HPV-1 + HPV-2^b^ |

DT(aP): diphtheria-tetanus-(acellular pertussis) vaccine; HepB: hepatitis B vaccine; Hib: *Haemophilus influenzae* type b vaccine; HPV: human papillomavirus vaccine (2 serotypes); IPV: inactivated polio vaccine; MenC: meningococcal C-conjugate vaccine; MMR: measles-mumps-rubella vaccine; PCV: pneumococcal conjugate vaccine (10 serotypes).

^a^ Only for children whose mother tested positive for hepatitis B surface antigen (HBsAg).

^b^ Only for girls.

(Source: <http://rijksvaccinatieprogramma.nl>)

Figure S1 Opinion of parents on statements regarding vaccination in general

Figure S2 Perceived severity by parents of diseases currently included in the National Immunisation Programme plus varicella, rotavirus gastroenteritis, meningococcal B disease, and seasonal influenza

**References**

1. van Lier A, Oomen P, de Hoogh P, Drijfhout I, Elsinghorst B, Kemmeren J, et al. Praeventis, the immunisation register of the Netherlands: a tool to evaluate the National Immunisation Programme. Euro Surveill. 2012;17(17):pii=20153.

2. van Lier EA, Oomen PJ, Oostenbrug MW, Zwakhals SL, Drijfhout IH, de Hoogh PA, et al. Hoge vaccinatiegraad van het Rijksvaccinatieprogramma in Nederland. [High vaccination coverage of the National Immunization Programme in the Netherlands]. Ned Tijdschr Geneeskd. 2009;153(20):950-7. Dutch.

3. van Lier EA, Geraedts JLE, Oomen PJ, Giesbers H, van Vliet JA, Drijfhout IH, et al. Vaccinatiegraad en jaarverslag Rijksvaccinatieprogramma Nederland 2016. [Vaccination coverage and annual report National Immunisation Programme Netherlands 2016]. Bilthoven: National Institute for Public Health and the Environment (RIVM); 2017. RIVM report 2017-0010. Dutch.

4. Paulussen TG, Hoekstra F, Lanting CI, Buijs GB, Hirasing RA. Determinants of Dutch parents' decisions to vaccinate their child. Vaccine. 2006;24(5):644-51.

5. Harmsen IA. Vaccinating: self-evident or not? Development of a monitoring system to evaluate acceptance of the National Immunization Program. Doctoral Dissertation. Maastricht: Maastricht University / RIVM; 2014.

6. Lehmann BA, de Melker HE, Timmermans DRM, Mollema L. Informed decision making in the context of childhood immunization. Patient Educ Couns. 2017.

7. Spaan DH, Ruijs WLM, Hautvast JLA, Tostmann A. Increase in vaccination coverage between subsequent generations of orthodox Protestants in The Netherlands. Eur J Public Health. 2017;27(3):524-30.

8. Klomp JH, van Lier A, Ruijs WL. Vaccination coverage for measles, mumps and rubella in anthroposophical schools in Gelderland, The Netherlands. Eur J Public Health. 2015;25(3):501-5.

9. Health Council of the Netherlands. Het individuele, collectieve en publieke belang van vaccinatie. [The individual, collective and public importance of vaccination]. The Hague: Health Council of the Netherlands; 2013. publication no. 2013/21. Dutch.
