# Supplementary material for: Intention to vaccinate universally against varicella, rotavirus gastroenteritis, meningococcal B disease and seasonal influenza among parents in the Netherlands: an internet survey
Source: BMC Res Notes. 2017 Dec 4;10:672. doi: 10.1186/s13104-017-3004-z (PMC5716237; doi:10.1186/s13104-017-3004-z)
Supplement: Supplementary file 2 — Additional file 2. Internet survey. [file 13104_2017_3004_MOESM2_ESM.docx]

**Additional file 2 Internet survey**

**Questionnaire on vaccination**

The government provides a National Immunisation Programme (NIP) for all children in the Netherlands. With the NIP we protect children from severe infectious diseases. Continuous research is being carried out on new vaccines to immunise children against all kinds of diseases. Some of these new vaccines may be eligible for inclusion in the NIP in the future. However, before a new vaccine is included in the NIP, it is important to know if parents actually want to have their child(ren) vaccinated with such a new vaccine. The National Institute for Public Health and the Environment (RIVM) investigates this.

Explanation of the questionnaire

In this questionnaire we ask *your* opinion, so there are no correct or incorrect answers. We are interested in all opinions, so also if you do not vaccinate your child(ren), your opinion is important to us. First of all, we ask you some questions about your personal situation and how you think about vaccinations in general. Subsequently, we will ask you some questions about (vaccination against) a number of diseases that are not currently covered by the National Immunisation Programme (NIP). Finally, we will ask you a few questions about pertussis.

Would you like to complete this questionnaire as fully as possible? This will take about 20 minutes. Your answers will be processed confidentially and anonymously (your name and address will not be provided to the researcher), will not be used for purposes other than this research and not provided to third parties.

**PART 1: GENERAL QUESTIONS**

1. What date did you fill out this questionnaire?

 - - 2012

1. What is your respondent number? This number is on the bottom right of your invitation letter.

1. What is your gender?

🞏 Male

🞏 Female

1. What is your age?

🞏 Younger than 25 years

🞏 25-29 years

🞏 30-34 years

- 35-39 years
- 40 years or older

1. What are the four digits of your zip code?

1. What is your highest completed education?

🞏 No education (primary education not completed)

🞏 Primary education (primary school, special primary education)

🞏 Lower vocational education (such as ‘LTS’, ‘LEAO’, ‘LHNO’, ’VMBO’)

- Lower secondary education (such as ‘MAVO’, ‘(M)ULO’, ‘MBO-kort’, ‘VMBO-t’)
- Intermediate vocational education (such as ‘MBO-lang’, ’MTS’, ‘MEAO’, ‘BOL’, ‘BBL’, ‘INAS’)
- Higher secondary education (such as ‘HAVO’, ‘VWO’, ‘Atheneum’, ‘Gymnasium’, ‘HBS’, ‘MMS’)
- Higher vocational education (such as ‘HBO’, ‘HTS’, ‘HEAO’, ‘kandidaatswetenschappelijk onderwijs’)
- Scientific education (university)

1. What is your country of birth?

🞏 the Netherlands

🞏 Suriname

🞏 Netherlands Antilles

🞏 Aruba

🞏 Turkey

🞏 Morocco

- Another country, namely…………………………………………

1. What is the country of birth of your mother and father?

Your mother Your father

The Netherlands 🞏 🞏

Suriname 🞏 🞏

Netherlands Antilles 🞏 🞏

Aruba 🞏 🞏

Turkey 🞏 🞏

Morocco 🞏 🞏

Another country, namely ………………………… …………………………

1. What is the net monthly income of your household?

*Net is the amount you receive on your bank account on a monhtly basis. These include income from labor, pension, social welfare payment or alimony. Child allowance and holiday payment not included.*

- Less than € 850,-
- € 851,- up to and including € 1.150,-
- € 1.151,- up to and including € 1.750,-
- € 1.751,- up to and including € 3.050,-
- € 3.051,- up to and including € 3.500,-
- € 3.501,- or more
- I don’t want to answer

1. How many people does your household have (including yourself)?

*With household we mean a group of people living together, such as a family.*

 persons

1. How many children do you have?

 children

1. Are there any children in your household who visit a daycare centre?
    *One day part is half a day. Do you want to add the day parts in case of multiple children?*

🞏 Yes, namely day parts a week

🞏 No

**PART 2: QUESTIONS ABOUT VACCINATION IN GENERAL**

1. Have your child(ren) participated / do(es) your child(ren) participate in the National Immunisation Programme (NIP)?

- Yes, fully (=got all vaccinations they should have had for their age)
- Yes, partly (=did not get all vaccinations they should have had for their age)

🞏 No

🞏 I don’t know

1. Has your opinion on vaccination changed in the past 5 years?

🞏 No

🞏 Yes, now more inclined to vaccinate

🞏 Yes, now less inclined to vaccinate

🞏 I don’t know

1. Is your opinion on vaccination influenced by one of the following issues?
    🞏 Anthroposophical philosophy

🞏 Homeopathic philosophy

🞏 Alternative medicine

🞏 Religion

- Other, namely …………………………………………………………………………………………………………………………
- None of the above

1. Below are some statements. Would you like to indicate per statement to what extent you agree?
    *(pleas tick one answer per statement)*

Strongly Disagree Neutral Agree Strongly disagree agree

Childhood vaccinations are good for the protection of
the health of my child 🞏 🞏 🞏 🞏 🞏

There is no need for vaccinating healthy children against
childhood diseases 🞏 🞏 🞏 🞏 🞏

I have doubts about the safety of the vaccinations children
receive 🞏 🞏 🞏 🞏 🞏

The immune system of my child will be negatively affected
by vaccination 🞏 🞏 🞏 🞏 🞏

Childhood vaccinations are good for the health protection
of others 🞏 🞏 🞏 🞏 🞏

Vaccinating my child is a matter of course 🞏 🞏 🞏 🞏 🞏

Nowadays, too many vaccinations are administered to children 🞏 🞏 🞏 🞏 🞏

1. Would you like to give each of the following diseases a score between 1 and 10 which you think is best to indicate the severity of the disease? Choose 1 if you find the disease *not severe at all* and 10 of you find the disease *very severe*.

Not

severe Very

at all severe Don’t

1 2 3 4 5 6 7 8 9 10 know

Diphtheria 🞏 1 🞏 2 🞏 3 🞏 4 🞏 5 🞏 6 🞏 7 🞏 8 🞏 9 🞏 10 🞏

Pertussis 🞏 1 🞏 2 🞏 3 🞏 4 🞏 5 🞏 6 🞏 7 🞏 8 🞏 9 🞏 10 🞏

Tetanus 🞏 1 🞏 2 🞏 3 🞏 4 🞏 5 🞏 6 🞏 7 🞏 8 🞏 9 🞏 10 🞏

Poliomyelitis 🞏 1 🞏 2 🞏 3 🞏 4 🞏 5 🞏 6 🞏 7 🞏 8 🞏 9 🞏 10 🞏

Varicella 🞏 1 🞏 2 🞏 3 🞏 4 🞏 5 🞏 6 🞏 7 🞏 8 🞏 9 🞏 10 🞏

*Haemophilus influenzae* type b

(Hib)-infection 🞏 1 🞏 2 🞏 3 🞏 4 🞏 5 🞏 6 🞏 7 🞏 8 🞏 9 🞏 10 🞏

Pneumococcal disease 🞏 1 🞏 2 🞏 3 🞏 4 🞏 5 🞏 6 🞏 7 🞏 8 🞏 9 🞏 10 🞏

Cervical cancer (HPV) 🞏 1 🞏 2 🞏 3 🞏 4 🞏 5 🞏 6 🞏 7 🞏 8 🞏 9 🞏 10 🞏

Hepatitis B 🞏 1 🞏 2 🞏 3 🞏 4 🞏 5 🞏 6 🞏 7 🞏 8 🞏 9 🞏 10 🞏

Mumps 🞏 1 🞏 2 🞏 3 🞏 4 🞏 5 🞏 6 🞏 7 🞏 8 🞏 9 🞏 10 🞏

Measles 🞏 1 🞏 2 🞏 3 🞏 4 🞏 5 🞏 6 🞏 7 🞏 8 🞏 9 🞏 10 🞏

Rubella 🞏 1 🞏 2 🞏 3 🞏 4 🞏 5 🞏 6 🞏 7 🞏 8 🞏 9 🞏 10 🞏

Rotavirus gastroenteritis 🞏 1 🞏 2 🞏 3 🞏 4 🞏 5 🞏 6 🞏 7 🞏 8 🞏 9 🞏 10 🞏

Meningococcal C disease 🞏 1 🞏 2 🞏 3 🞏 4 🞏 5 🞏 6 🞏 7 🞏 8 🞏 9 🞏 10 🞏

Meningococcal B disease 🞏 1 🞏 2 🞏 3 🞏 4 🞏 5 🞏 6 🞏 7 🞏 8 🞏 9 🞏 10 🞏

Seasonal influenza 🞏 1 🞏 2 🞏 3 🞏 4 🞏 5 🞏 6 🞏 7 🞏 8 🞏 9 🞏 10 🞏

**PART 3: QUESTIONS ABOUT VACCINATION AGAINST ROTAVIRUS GASTROENTERITIS, MENINGOCOCCAL B DISEASE, SEASONAL INFLUENZA AND VARICELLA**

Within the current Nationale Immunisation Programme (NIP) there is no vaccination against rotavirus gastroenteritis, meningococcal B disease, seasonal influenza or varicella. However, vaccines against these diseases have been developed or are still under development. The following questions are about your opinion on vaccination against these diseases. First, we give a brief description of the general disease symptoms. For all these diseases hospitalisation is sometimes necessary and a child may die.

**ROTAVIRUS GASTROENTERITIS**

Usually starts with nausea and vomiting, followed by diarrhoea. After a few days the vomiting stops, after which the diarrhoea diminishes slowly. Known complications include dehydration.

**MENINGOCOCCAL DISEASE**

Sudden high fever, chills, muscle aches, and vomiting followed by headache. Known complications are meningitis and blood poisoning.

**SEASONAL INFLUENZA**

Influenza is usually characterized by (1) an acute onset, (2) cough, rhinitis, sneezing, pain behind the breastbone, sore throat, and (3) fever up to 39°C or higher, joint pain, headache, cold chills, malaise, fatigue and muscle pain. Known complications include ear infections and pneumoniae.

**VARICELLA**

A few days of fever and rash starting on the head or torso: small bumps that develop into blisters that may itch considerably. Known complications include skin infections, febrile convulsions and encephalitis.

1. Would you like to indicate for each of the following diseases whether you would vaccinate your own child(ren) if the vaccination is included in the National Immunisation Programme (NIP)? All vaccinations within the National Immunisation Programme (NIP) are free of charge. (INTENTION A)

Yes, Yes, Maybe yes, No, No, definitely probably maybe no probably not definitely not

Rotavirus gastroenteritis 🞏 🞏 🞏 🞏 🞏

Meningococcal B disease 🞏 🞏 🞏 🞏 🞏

Seasonal influenza 🞏 🞏 🞏 🞏 🞏

Varicella 🞏 🞏 🞏 🞏 🞏

1. Would you like to indicate for each of the following diseases whether you would vaccinate your own child(ren) if you have to pay for the vaccination yourself? (INTENTION B)

Yes, Yes, Maybe yes, No, No, definitely probably maybe no probably not definitely not

Rotavirus gastroenteritis 🞏 🞏 🞏 🞏 🞏

Meningococcal B disease 🞏 🞏 🞏 🞏 🞏

Seasonal influenza 🞏 🞏 🞏 🞏 🞏

Varicella 🞏 🞏 🞏 🞏 🞏

**ROTAVIRUS GASTROENTERITIS**: *Usually starts with nausea and vomiting, followed by diarrhoea. After a few days the vomiting stops, after which the diarrhoea diminishes slowly. Known complications include dehydration.*

1. Below some statements on vaccination against **rotavirus gastroenteritis**. Would you like to indicate to what extent you agree with each of these statements? *(please tick 1 answer per statement)*

Strongly Strongly disagree Disagree Neutral Agree agree

I think it is important to vaccinate my child against

rotavirus gastroenteritis (ATTITUDE) 🞏 🞏 🞏 🞏 🞏

Rotavirus gastroenteritis is a severe disease
(RISK PERCEPTION: DISEASE) 🞏 🞏 🞏 🞏 🞏

I think rotavirus gastroenteritis is a disease severe enough

to vaccinate against (RISK PERCEPTION: CONDITIONAL VACCINATION) 🞏 🞏 🞏 🞏 🞏

I think it is very likely that my child will get rotavirus

gastroenteritis (RISK PERCEPTION: CHANCE) 🞏 🞏 🞏 🞏 🞏

I think most parents will vaccinate their child against

rotavirus gastroenteritis (SUBJECTIVE NORM – DESCRIPTIVE NORM) 🞏 🞏 🞏 🞏 🞏

Most people who are important to me will approve of my

vaccinating my child against rotavirus gastroenteritis
(SUBJECTIVE NORM – INJUNCTIVE NORM) 🞏 🞏 🞏 🞏 🞏

I am worried about the side effects of vaccination against

rotavirus gastroenteritis (OUTCOME EXPECTATION) 🞏 🞏 🞏 🞏 🞏

I like the rotavirus vaccine being orally administered and

therefore no additional vaccination is needed 🞏 🞏 🞏 🞏 🞏

**MENINGOCOCCAL DISEASE**: *Sudden high fever, chills, muscle aches, and vomiting followed by headache. Known complications are meningitis and blood poisoning.*

1. Below some statements on vaccination against **meningococcal B disease**. Would you like to indicate to what extent you agree with each of these statements? *(please tick 1 answer per statement)*

Strongly Strongly disagree Disagree Neutral Agree agree

I think it is important to vaccinate my child against

meningococcal B disease (ATTITUDE) 🞏 🞏 🞏 🞏 🞏

Meningococcal B disease is a severe disease
(RISK PERCEPTION: DISEASE) 🞏 🞏 🞏 🞏 🞏

I think meningococcal B disease is a disease severe enough

to vaccinate against (RISK PERCEPTION: CONDITIONAL VACCINATION) 🞏 🞏 🞏 🞏 🞏

I think it is very likely that my child will get

meningococcal B disease (RISK PERCEPTION: CHANCE) 🞏 🞏 🞏 🞏 🞏

I think most parents will vaccinate their child against

meningococcal B disease (SUBJECTIVE NORM – DESCRIPTIVE NORM) 🞏 🞏 🞏 🞏 🞏

Most people who are important to me will approve of my

vaccinating my child against meningococcal B disease
(SUBJECTIVE NORM – INJUNCTIVE NORM) 🞏 🞏 🞏 🞏 🞏

I am worried about the side effects of vaccination against

meningococcal B disease (OUTCOME EXPECTATION) 🞏 🞏 🞏 🞏 🞏

**SEASONAL INFLUENZA**: *Influenza is usually characterized by (1) an acute onset, (2) cough, rhinitis, sneezing, pain behind the breastbone, sore throat, and (3) fever up to 39°C or higher, joint pain, headache, cold chills, malaise, fatigue and muscle pain.
Known complications include ear infections and pneumoniae.*

1. Below some statements on vaccination against **seasonal influenza in children**. This question does not concern flu vaccination in elderly. Would you like to indicate to what extent you agree with each of these statements? *(please tick 1 answer per statement)*

Strongly Strongly disagree Disagree Neutral Agree agree

I think it is important to vaccinate my child against

seasonal influenza (ATTITUDE) 🞏 🞏 🞏 🞏 🞏

Seasonal influenza is a severe disease
(RISK PERCEPTION: DISEASE) 🞏 🞏 🞏 🞏 🞏

I think seasonal influenza is a disease severe enough

to vaccinate against (RISK PERCEPTION: CONDITIONAL VACCINATION) 🞏 🞏 🞏 🞏 🞏

I think it is very likely that my child will get seasonal influenza
(RISK PERCEPTION: CHANCE) 🞏 🞏 🞏 🞏 🞏

I think most parents will vaccinate their child against

seasonal influenza (SUBJECTIVE NORM – DESCRIPTIVE NORM) 🞏 🞏 🞏 🞏 🞏

Most people who are important to me will approve of my

vaccinating my child against seasonal influenza
(SUBJECTIVE NORM – INJUNCTIVE NORM) 🞏 🞏 🞏 🞏 🞏

I am worried about the side effects of vaccination against

seasonal influenza (OUTCOME EXPECTATION) 🞏 🞏 🞏 🞏 🞏

**VARICELLA**: *A few days of fever and rash starting on the head or torso: small bumps that develop into blisters that may itch considerably. Known complications include skin infections, febrile convulsions and encephalitis.*

1. Below some statements on vaccination against **varicella**. Would you like to indicate to what extent you agree with each of these statements? *(please tick 1 answer per statement)*

Strongly Strongly disagree Disagree Neutral Agree agree

I think it is important to vaccinate my child against varicella
(ATTITUDE) 🞏 🞏 🞏 🞏 🞏

Varicella is a severe disease (RISK PERCEPTION: DISEASE) 🞏 🞏 🞏 🞏 🞏

I think varicella is a disease severe enough to vaccinate against
(RISK PERCEPTION: CONDITIONAL VACCINATION) 🞏 🞏 🞏 🞏 🞏

I think it is very likely that my child will get varicella
(RISK PERCEPTION: CHANCE) 🞏 🞏 🞏 🞏 🞏

I think most parents will vaccinate their child against varicella
(SUBJECTIVE NORM – DESCRIPTIVE NORM) 🞏 🞏 🞏 🞏 🞏

Most people who are important to me will approve of my

vaccinating my child against varicella (SUBJECTIVE NORM –
INJUNCTIVE NORM) 🞏 🞏 🞏 🞏 🞏

I am worried about the side effects of vaccination against

varicella (OUTCOME EXPECTATION) 🞏 🞏 🞏 🞏 🞏

*Additional questions on (vaccination against) varicella and pertussis from the original questionnaire are not included in this translated version because the results of these questions are not discussed in the main text.*

1. Would you like to re-enter your respondent number for verification? This number is on the bottom right of your invitation letter.

1. Do you have any comments about the questionnaire or the research?

…………………………………………………………………………………………………………………………………………..

…………………………………………………………………………………………………………………………………………..

…………………………………………………………………………………………………………………………………………..

…………………………………………………………………………………………………………………………………………..

…………………………………………………………………………………………………………………………………………..

…………………………………………………………………………………………………………………………………………..

…………………………………………………………………………………………………………………………………………..

…………………………………………………………………………………………………………………………………………..

…………………………………………………………………………………………………………………………………………..

…………………………………………………………………………………………………………………………………………..

…………………………………………………………………………………………………………………………………………..

…………………………………………………………………………………………………………………………………………..

…………………………………………………………………………………………………………………………………………..

…………………………………………………………………………………………………………………………………………..

…………………………………………………………………………………………………………………………………………..

…………………………………………………………………………………………………………………………………………..

…………………………………………………………………………………………………………………………………………..

…………………………………………………………………………………………………………………………………………..

**This is the end of the questionnaire.**

**Thank you for your cooperation!**

**For your cooperation you will receive a 10 euro voucher by January 2013 at latest.**
